# Profiles of microRNA networks in intestinal epithelial cells in a mouse model of colitis

**DOI:** 10.1038/srep18174

**Published:** 2015-12-09

**Authors:** Juneyoung Lee, Eun Jeong Park, Yoshikazu Yuki, Shandar Ahmad, Kenji Mizuguchi, Ken J. Ishii, Motomu Shimaoka, Hiroshi Kiyono

**Affiliations:** 1 Division of Mucosal Immunology, Department of Microbiology and Immunology, The Institute of Medical Science, The University of Tokyo, Tokyo 108-8639, Japan; 2 International Research and Development Center for Mucosal Vaccine, The Institute of Medical Science, The University of Tokyo, Tokyo 108-8639, Japan; 3 Department of Computational Biology and Medical Sciences, Graduate School of Frontier Sciences, The University of Tokyo, Chiba 277-8561, Japan; 4 Laboratory of Bioinformatics, National Institute of Biomedical Innovation, Osaka 567-0085, Japan; 5 Laboratory of Adjuvant Innovation, National Institute of Biomedical Innovation, Osaka 567-0085, Japan; 6 Laboratory of Vaccine Science, Immunology Frontier Research Center, World Premier Institute, Osaka University, Osaka 565-0871, Japan; 7 Department of Molecular Pathobiology and Cell Adhesion Biology, Mie University Graduate School of Medicine, Mie 514-8507, Japan; 8 Core Research for Evolutional Science and Technology (CREST), Japan Science and Technology Agency, Tokyo 102-0075, Japan

## Abstract

Inflammatory bowel diseases (IBDs) accompany a critical loss of the frontline barrier function that is achieved primarily by intestinal epithelial cells (IECs). Although the gene-regulation pathways underlying these host-defense roles of IECs presumably are deranged during IBD pathogenesis, the quantitative and qualitative alterations of posttranscriptional regulators such as microRNAs (miRNAs) within the cells largely remain to be defined. We aimed to uncover the regulatory miRNA–target gene relationships that arise differentially in inflamed small- compared with large-IECs. Whereas IBD significantly increased the expression of only a few miRNA candidates in small-IECs, numerous miRNAs were upregulated in inflamed large-IECs. These marked alterations might explain why the large, as compared with small, intestine is more sensitive to colitis and shows more severe pathology in this experimental model of IBD. Our in-depth assessment of the miRNA–mRNA expression profiles and the resulting networks prompts us to suggest that miRNAs such as miR-1224, miR-3473a, and miR-5128 represent biomarkers that appear in large-IECs upon IBD development and co-operatively repress the expression of key anti-inflammatory factors. The current study provides insight into gene-regulatory networks in IECs through which dynamic rearrangement of the involved miRNAs modulates the gene expression–regulation machinery between maintaining and disrupting gastrointestinal homeostasis.

IBD is a chronic relapsing disorder of the gastrointestinal tract that is due to intestinal inflammation and epithelial injury and includes Crohn’s disease and ulcerative colitis. Although IBD is thought to arise through interactions among genetic, immunologic, and environmental factors, the etiology underlying the pathophysiology of IBD remains largely unknown. In particular, Crohn’s disease and ulcerative colitis appear to result from distinct pathologic mechanisms that have a shared origin in the deterioration of the gastrointestinal barrier function, which is supported primarily by IECs and their secreted products^1^. In addition to their role as a physical barrier, IECs produce pro-inflammatory chemokines and cytokines in response to luminal stimuli and recruit diverse immune cells in the lamina propria to inflamed regions in the gut^2^. Thus IECs are at the frontline of the host immune defense system and afford a continuous response to internal and external stimuli under inflamed as well as normal conditions. In this way, IECs are integral to both innate and adaptive immunity as well as the maintenance of gastrointestinal homeostasis. Analyzing the genetic repertoire of primary IECs is therefore a fundamental step in investigating the etiologic factors and interactions involved in the development of IBD.

miRNAs are a class of endogenous, small noncoding RNA molecules that control gene expression at the posttranscriptional level^3^. Considerable attention has been given to identifying unique miRNA expression profiles that are putatively implicated in either aggravating or ameliorating IBD^4^. Investigations of the miRNA profiles associated with IBD have developed our understanding of their involvement in IBD pathogenesis. However, most studies of miRNA profiling in intestines have used whole-tissue preparations, which present limitations regarding cell-based analysis of miRNA expression. First, intestinal tissue comprises the functionally and anatomically distinct layers of the mucosa, submucosa, and muscularis externa; furthermore the mucosa itself comprises multiple cell types, including lymphocytes, monocytes, and enterocytes. Second, whole-tissue intestinal preparations might include various luminal components, such as residual secretions, microflora, and their products. Considering the importance of the phenotypic plasticity of IECs in the gastrointestinal tract, comprehensively examining the miRNA expression profiles of inflamed and normal IECs is a first step in identifying IBD-induced miRNA biomarkers in IECs.

The phenotypic and anatomic differences between ulcerative colitis and Crohn’s disease are thought to reflect the intestinal region affected: ulcerative colitis is limited to the colon, whereas Crohn’s disease can occur throughout the gastrointestinal tract^5^. Among animal models, dextran sodium sulfate (DSS)-induced colitis in mice resembles the damage manifested in patients with ulcerative colitis and displays clinical signs, including rectal bleeding, weight loss, and diarrhea, that are common characteristics of the human disease^6^. Given the differing sensitivities of the small and large intestines to DSS-induced inflammation, it will be of significance to find any reliable source for the regional difference through obtaining IEC-specific, global regulation network at the genetic basis. Furthermore, by sorting for and analyzing only live IECs, the potential confounding effect of miRNAs and mRNAs from dead cells on the expression patterns of live cells can be minimized.

Here, we used IECs isolated from mice with and without DSS-induced colitis to: 1) determine the hierarchical profiles of miRNAs and mRNA expression in small- compared with large-IECs, and 2) propose associations between miRNAs and their target genes that explain the differing sensitivities and pathologic outcomes of the small compared with large intestine to DSS-induced colitis. Finally, we suggest gene regulatory networks in IECs that link miRNAs and targets in IBD and provide a strategy for identifying biomarkers and targets that might improve IBD diagnosis and treatment.

## Results

### Small and large intestines respond differently to experimentally induced colitis

We used the DSS-induced colitis model, in which the disease symptoms of affected mice are similar to those in human patients with ulcerative colitis^6^. According to this model, Balb/c mice received 3% DSS in drinking water for 8 d to induce gut inflammation; whereas treated mice had a marked loss in body weight, untreated mice gained weight normally over time (Supplementary Figure 1a). In addition, DSS-treated mice euthanized on day 8 had bloody stools and significant reduction in the length of their large, but not small, intestine, consistent with colitis development (Supplementary Figure 1b). Furthermore, whereas the small intestine of DSS-treated mice showed only minor histologic changes, their large intestine displayed symptoms such as submucosa edema, decreased crypt depth, and the infiltration of inflammatory leukocytes (Supplementary Figure 1c). Therefore, among the mice treated with DSS, the symptoms in the large intestine were much more severe than those in the small intestine. These results are consistent with previous finding^7^ and show the reliability of the model for use in the IEC miRNA profiling study.

### The miRNA expression profile differs between small- and large-IECs under physiologic conditions

To obtain comprehensive profiles of the miRNAs that are expressed differentially in the epithelial cells of the small intestine compared with the large intestine under physiologic conditions, we first isolated IECs from the intestinal tissue of DSS-untreated mice. Because the isolation protocol yields heterogeneous cell populations, we used flow cytometry to sort the IECs according to cell size. Using large-IECs as an example, the cell population indicated as “iii” (Supplementary Figure 2a) largely excluded cells positive for CD45 (mononuclear cells, i) and Via-Probe (dead cells, ii). In addition, we confirmed that cells from population “iii” were negative for CD45 but positive for epithelial cell adhesion molecule and cytokeratin (Supplementary Figure 2b). Next, we collected only Via-Probe-negative, live IECs (shaded area) for use in RNA purification. We then surveyed the miRNA microarray by using RNAs purified from the sorted cells and analyzed their expression levels in small- and large-IECs.

miRNAs were selected according to their expression intensity in normal small- compared with large-IECs. Of approximately 1,900 miRNAs on the microarray, normal small-IECs contained 86 miRNAs whose expression was markedly increased in terms of both fold change (>2-fold) and fluorescence intensity (>100 units above background), whereas 33 miRNAs in normal large-IECs showed increased expression (Table 1). In addition, the expression of 9 miRNAs in small-IECs (miR-6243, -5109, -2137, -6240, -5126, -3960, -2861, -711, and -762) but none in large-IECs was highly increased (>2-fold induction and fluorescence intensity >10,000) (Table 1). By contrast, both small- and large-IECs contained 4 ~ 500 miRNAs whose expression was below the background level (data not shown). These results indicate that under physiologic conditions, small-IECs had more miRNAs that met our criteria for increased expression than did large-IECs.

### The miRNA expression profile differs between small- and large-IECs from mice with DSS-induced colitis

Using methodology similar to that for assessing miRNA expression under physiologic conditions, we evaluated the miRNA profiles of small- and large-IECs harvested from DSS-treated mice. The miRNA expression profiles of inflamed small- and large-IECs exhibited striking differences from those of normal small- and large-IECs (Table 1). In particular, the small-IECs isolated from DSS-treated mice had far fewer highly expressed miRNAs (13 miRNAs) than did those from untreated mice (86 miRNAs). All 13 of the highly expressed miRNAs from inflamed small-IECs overlapped those highly expressed in the small-IECs of untreated mice. This finding indicates that the inflamed condition did not induce any miRNA in small-IECs in addition to those present under physiologic conditions; in other words, DSS-induced inflammation down-regulated the overall miRNA expression level in small-IECs.

By contrast, 39 miRNAs in the large-IECs of mice with DSS-induced colitis showed increased expression (Table 1). Interestingly, these 39 large-IEC miRNAs comprised three distinct groups: 10 newly identified miRNAs, 18 miRNAs that overlapped with those highly expressed in normal small-IECs, and 11 miRNAs that overlapped with those highly expressed in normal large-IECs. Moreover, inflamed large-IECs yielded 3 miRNAs (miR-5126, -690, and -5105) whose expression was highly increased (>2-fold induction and fluorescence intensity >10,000) (Table 1). Therefore, these results suggest that small- and large-IECs display unique patterns of miRNA expression under physiologic compared with inflamed conditions. Categorizing the miRNA biomarkers expressed in each IEC compartment might provide insight into the differential sensitivity to and pathologic severity of DSS-induced inflammation in the small compared with large intestine.

### Comparing the global miRNA profiles reveals the expression modules unique to small compared with large inflamed IECs

**Figure 1:**
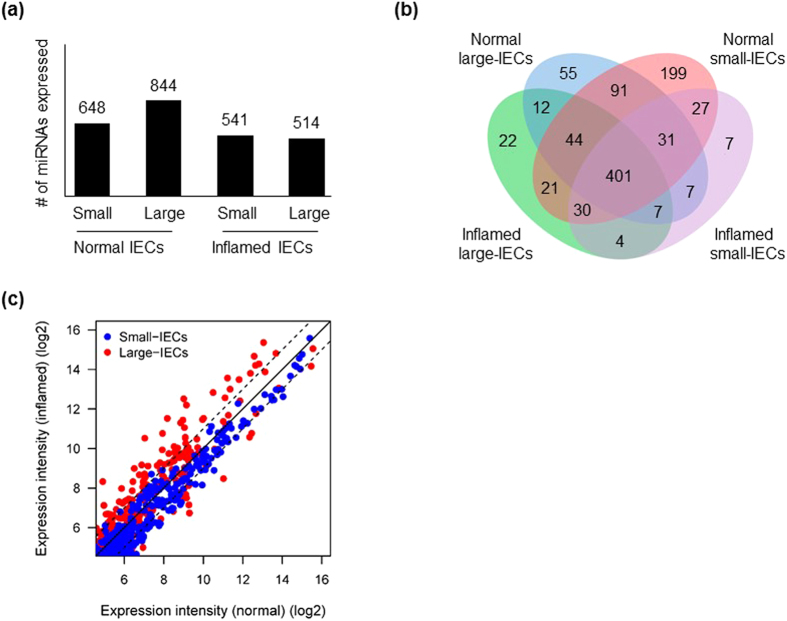
Schematic diagrams of similarity or overlap of miRNA populations among four assay groups: normal small-IECs, normal large-IECs, inflamed small-IECs, and inflamed large-IECs. (**a**) The total numbers of miRNAs in each group are shown in the bar graph. (**b**) Venn diagram comparing miRNA expression in normal large-, normal small-, inflamed large-, and inflamed small-IECs. (**c**) Scatterplot showing total miRNA expression. Expression intensity values were pooled, and global median values were computed. The central diagonal line indicates equivalence of expression between the *x* and *y* axes. The 2 diagonal dotted lines indicate a 2-fold up- or down-regulation difference.

We next examined overall numbers of miRNAs highly expressed in each experimental group (normal small-, inflamed small-, normal large-, and inflamed large-IECs). We set the expression threshold as the median expression value of all four miRNA arrays and analysed the population overlaps. Overall, 648, 844, 541, and 514 miRNAs were expressed in normal small-IECs, normal large-IECs, inflamed small-IECs, and inflamed large-IECs, respectively (Fig. 1a), and 401 miRNAs appear to be common to all groups, thus reflecting a non-specific miRNA population characteristic of IECs in general (Fig. 1b). A total of 233 miRNAs was expressed exclusively in small-IECs (both normal and inflamed) compared with 89 miRNAs in large-IECs, indicating the larger miRNA population in small-IECs. However, 199 of the 233 miRNAs were unique to normal small-IECs compared with 55 among the 89 miRNAs restricted to large-IECs (that is, almost four-fold more in small- versus large-IECs). In contrast, inflamed small-IECs yielded 7 unique miRNAs versus 22 in inflamed large-IECs (about three-fold more in large-IECs).

We then compared the global miRNA expression level between small- and large-IECs from DSS-treated mice. Numerous highly expressed miRNAs in the large-IECs were increased in the inflamed, compared with normal, condition (Fig. 1c). Conversely, miRNAs in small-IECs were remarkably up-regulated in the normal, compared with inflamed, condition. These data suggest that many more miRNAs in large-IECs robustly respond to the inflammatory situation compared with those in small-IECs.

### The different patterns of miRNA expression in IECs under normal and inflamed conditions indicate the differing susceptibility to and severity of DSS-induced inflammation in the small compared with large intestine

We attempted to acquire a profile of the IEC miRNAs by measuring their fold change after DSS administration. The criteria for choosing miRNAs of interest was identical to that used to compare miRNAs between small- and large-IECs in Table 1 and a previous report^8^. The small-IECs of the mice with DSS-induced colitis contained 1 miRNA (miR-223-3p) whose expression was highly increased and 29 miRNAs whose expression was highly decreased when compared with those of normal mice (Table 2). By contrast, the DSS-induced colitis condition caused large-IECs to express 51 miRNAs whose expression was highly increased and 15 miRNAs whose expression was highly decreased (Table 2). These results suggest that the DSS-induced inflammatory condition differentially modifies the small- and large-IEC miRNA profiles and that large-IECs exhibit a broader spectrum of miRNA expression.

**Figure 2:**
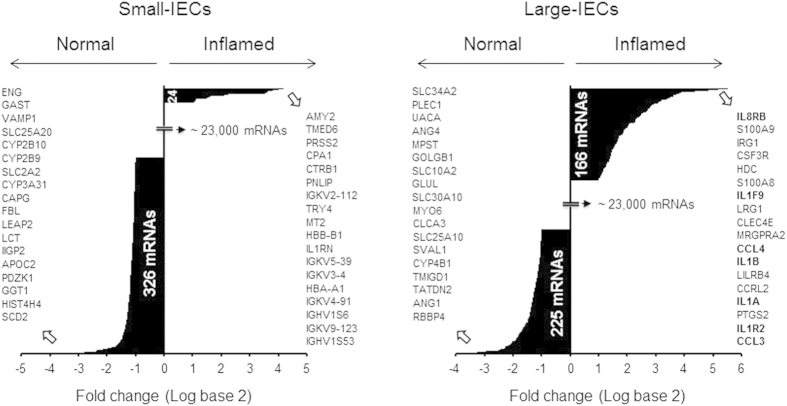
Hierarchy of mRNAs up- or down-regulated in inflamed, compared with normal, IECs. DSS-induced colitis led to the down-regulation of 326 mRNAs in small-IECs and 225 in large-IECs. By contrast, 24 and 166 genes were significantly up-regulated in inflamed small- and large-IECs, respectively. However, most genes (approximately 98.5%) failed to meet the selection criteria (>2-fold change and fluorescence intensity >100). The genes corresponding to the 18 mRNAs most highly up- (right) or down-regulated (left) in the inflamed, compared with normal, IECs are listed; genes for chemokines, cytokines, or their related molecules that were highly up-regulated in inflamed IECs are in bold face.

Again using the same selection criteria (that is, >2-fold induction and fluorescence intensity >100 arbitrary units above background), we analyzed mRNA microarrays with the small- and large-IEC samples from DSS-treated and untreated mice to acquire global gene-expression profiles in terms of fold change. Among the mRNAs of approximately 24,000 genes (Methods), the expression of 24 mRNAs was increased and that of 326 was decreased in inflamed small-IECs (Fig. 2). By contrast, the expression of 166 mRNAs was increased and that of 225 was decreased in inflamed large-IECs (Fig. 2). These data suggest that more mRNAs showed increased expression in inflamed, compared with normal, large-IECs than in small-IECs. For both small- and large-IECs, the 18 genes showing the greatest change in mRNA expression after the induction of inflammation are shown in Fig. 2. In particular, 7 of the 18 genes most highly up-regulated in inflamed large-IECs corresponded to chemokines, cytokines, or related molecules, thus indicating that large-IECs are the intestinal cell population that produces pro-inflammatory factors in response to inflammatory stimuli.

### The networks suggested by the inverse expression patterns of IEC miRNAs and their corresponding mRNAs reflect the different responsiveness of the small versus large intestine to DSS-induced inflammation

The miRNAs whose expression changed dramatically after DSS-induced colitis may contribute to pathogenesis by controlling the expression of their target genes. We therefore defined networks of miRNAs and their putative target genes in inflamed IECs in which the ‘direction’ of miRNA expression was opposite to that of the target gene. Considering that changes in miRNA-induced transcription repression might lead to altered gene expression, we created putative networks that comprised the miRNAs markedly up- or down-regulated in inflamed small- or large-IECs (Table 2) and their target mRNAs whose expression was dramatically decreased or increased, respectively, in the same cell population. We used the TargetScan database^9^ to identify putative target genes of miRNAs.

**Figure 3:**
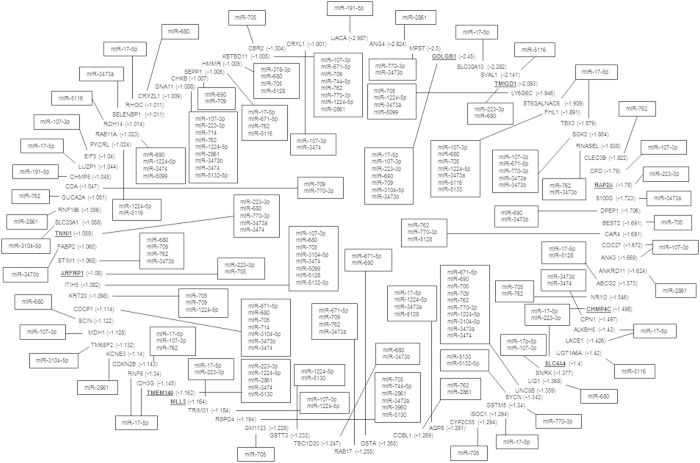
The miRNA-regulated interactions in inflamed large-IECs. The predicted interactions between miRNAs and target genes in large-IECs were visualized according to mutually inversely correlated expressions. Potential miRNA–mRNA regulatory interactions were shown according to the criteria used in this study (>2-fold induction and fluorescence intensity >100 arbitrary units above background). In inflamed large-IECs, 27 up-regulated miRNAs appear to regulate the expression of 83 mRNA targets. Multiple types of interactions between miRNAs and mRNA targets arise, including single-to-single, single-to-multiple, multiple-to-single, or multiple-to-multiple, thus indicating the diverse modes by which miRNAs might regulate gene expression. The numbers in parentheses indicate the fold change (log base 2) of the genes in inflamed, compared with normal, IECs; the putative target genes are listed clockwise in order of their fold change, and the single and multiple miRNAs that interact with various mRNAs are depicted along the outside and inside of the circle of mRNA targets, respectively. Associations were based on a TargetScan database analysis.

This process revealed that small-IECs harbored only one up-regulated miRNA (miR-223-3p) (Supplementary Figure 3a) and one down-regulated miRNA (miR-671-5p) (Supplementary Figure 3b) whose putative targets were simultaneously decreased (13 mRNAs) and increased (one mRNA), respectively, in the inflamed condition. In contrast, large-IECs exhibited numerous association pathways for the miRNAs showing altered expression in the inflamed condition (Fig. 3). We plotted the putative miRNA–mRNA regulatory networks inferred from their individual expression levels and computationally predicted target associations. The miRNA–target gene associations for the miRNAs up-regulated in inflamed large-IECs are shown in Fig. 3; those for down-regulated miRNA in inflamed large-IECs are shown in Supplementary Figure 4, respectively. Consistent with our other findings, the miRNAs with increased expression in inflamed large-IECs were involved in more putative regulatory networks than were those showing decreased expression in the same cells. These regulatory interactions might cause the post-transcriptional suppression of target genes in inflamed (Fig. 3) and normal (Supplementary Figure 4) large-IECs. Specifically, 27 miRNAs up-regulated in large-IECs are expected to target 83 mRNAs either individually or via multiple and redundant relationships (Fig. 3). In contrast, 13 miRNAs that are down-regulated in inflamed large-IECs may thus become incapable of regulating their 45 target genes, leading to the increased expression of these genes in the same cells (Supplementary Figure 4). Together, these results strongly suggest that an array of miRNAs in the IECs of the large intestine leads to large-scale target-gene silencing upon DSS-induction of experimental colitis.

Next, we further analyzed biological pathways possibly associated with the target genes of significantly altered miRNAs in inflamed large-IECs, by using a bioinformatics tools (TargetMine)^10^. We have selected significantly enriched 15 pathways in which more than two genes are associated (Supplementary Figure 5). Interestingly, among total 15 pathways enriched in inflamed large-IECs, 3 pathways were associated in platelet function, which reached 20%. Thus, these data could speculate that inflamed large-IECs possess a potential to modify platelet function presumably by augmenting any pro-inflammatory mediator or signaling molecule(s).

### The miR-223-3p in inflamed IECs may control the expression of different gene targets in the small versus large intestine

We then examined the putative target genes of miRNAs that were down- or up-regulated in inflamed IECs. In the microarray, the expression of miR-223-3p was augmented in inflamed, compared with normal, small-IECs. Consistent with this finding, miR-223-3p is one of the main miRNAs increased during IBD pathogenesis^11^. The expression of miR-223-3p was increased markedly in inflamed, compared with normal, IECs of both the small and large intestine (Table 2).

miR-223-3p might control the expression of its 13 putative targets, which were decreased in expression in inflamed small-IECs (Supplementary Figure 3a). Among these targets, DUOXA2 is known to produce hydrogen peroxide in human colon, and its expression is augmented in active colitis^12^. Furthermore, mice with macrophage-specific deletion of HSP90B1 (a target of miR-223-3p) are more resistant to DSS-induced colitis than are their wild-type counterparts^13^. In contrast, another target for miR-223-3p is angiotensin converting enzyme-2 (ACE2), which is required to express an amino acid transporter on the luminal surface of IECs; ACE2 appears to promote the development of gut inflammation because its deficiency decreases the severity of DSS-induced colitis^14^. Therefore, various putative targets for miR-223-3p that are down-regulated in inflamed small-IECs likely participate in either antagonizing (DUOXA2 or HSP90B1) or accelerating (ACE2) inflammatory responses.

Inflamed large-IECs contained 9 putative target genes for miR-223-3p whose expression was decreased in the same cells (Fig. 3): GOLGB1, TMIGD1, CHMP4C, SLC4A4, MLL3, TMEM140, ARFRP1, and TNNI1. Except for SLC4A4, these putative targets did not overlap with those decreased in inflamed small-IECs (Supplementary Figure 3a). Among the putative target genes for the large-IEC miR-223-3p, TMIGD1 expression is limited to the upper portion of the epithelial crypts in normal colorectal mucosa and likely is involved in IEC differentiation^15^. In addition, another miR-223-3p target, ARFRP1, seems to be necessary for lipid formation in the Golgi apparatus of IECs^16^. Therefore, some targets of miR-223-3p that are down-regulated in the inflamed condition might be involved in the differentiation or metabolism of large-IECs, suggesting a novel role of miR-223-3p in limiting lipid metabolism and cell growth in large-IECs during abnormal situations such as inflammation. Our findings indicate that miR-223-3p represses the gene expression of nearly exclusive targets in inflamed small- (92.3% unique targets) and large- (88.9%) IECs. In addition, this miRNA seems to specifically direct the gene-silencing machinery of the small compared with large intestine by targeting different genes during IBD development. This mechanism conceivably contributes to the apparent differential susceptibility of the small versus large intestine to DSS-induced inflammation (Supplementary Figure 1).

### The predicted networks of miRNA-coordinated gene regulation in the inflamed large-IECs imply a molecular-level mechanism for the differential susceptibility and pathology of the large intestine in DSS-induced inflammation

The population of miRNAs in inflamed large-IECs whose expression was markedly changed from that in normal IECs was largely different from those in inflamed small-IECs. To validate the results from the microarray analysis (Table 2), we used quantitative real-time PCR analysis of the representative miRNAs to reveal significantly increased (Supplementary Figure 6a) or decreased (Supplementary Figure 6b) miRNA expression in the inflamed, compared with normal, large-IECs. miRNAs with increased expression in inflamed large-IECs included miR-223-3p, -671-5p, -680, -690, -709, -1224-5p, -3473a, -3473b, -5116, -5128, and -5130; miRNAs with decreased expression were: let-7b-, -7c-, -7f-, and -7g-5p, and miR-21a-5p, -141-3p, -3963, and -3968. In case of the miRNAs with changed expression in inflamed, compared with normal, small-IECs according to microarray analysis, expression of miR-223-3p was higher in inflamed, compared with normal, small-IECs; the expression of miR671-5p was also up-regulated in inflamed small-IECs (Supplementary Figure 6c), which was discrepant with the microarray data.

We next sought to identify candidate targets for the miRNAs with significantly changed expression levels in both microarray and quantitative PCR analyses. Among the target genes whose expression was inversely proportional to that of miRNAs in the inflamed large-IECs, we selected genes of interest on the basis of their mention in previous studies. As shown in Table 3, genes with increased expression in inflamed large-IECs were categorized functionally into those associated with IBD, other inflammatory conditions, cancer, and gut immunity or homeostasis. The target genes associated with IBD include CCL3^17^, TREM1^18^, NCF4^19^, CXCL9^20^, NCF2^21^, SLC11A1^22^, CD97^23^, SFPQ^24^, and CXCL16^25^. The target genes involved in other inflammatory conditions include LILRB4^26^, CLEC4D^27^, FGR^28^, TGM2^29^, and IL17RA^30^. The cancer-related genes are IL4RA^31^ and CTSC^32^, and, the target genes involved in gut homeostasis or immunity are CLEC4E^33^, and PTPRC^34^. Of these candidate targets, 15 mRNAs shown in underline were validated for their significantly up-regulated expression by quantitative PCR analysis (Table 3 and Supplementary Figure 7a).

Next we analyzed the target genes that were down-regulated in inflamed large-IECs and the miRNAs whose expression was up-regulated; the expression levels of these miRNAs were validated through quantitative PCR analysis (Supplementary Figures 6a and 7b). The target genes of interest in this analysis (Table 4) that were related to IBD include ABCG2^35^, AQP8^36^, and SELENBP1^37^; those involved in other inflammatory processes include CAR4^38^, CPN1^39^, UNC5B^40^, SCIN^41^, STIM1^42^, and HMMR^43^. The cancer-related genes are RNASEL^44^, DPEP1^45^, MLL3^46^, CDCP1^47^, and KRT20^48^; those involved in gut homeostasis or immunity are TMIGD1^15^, ARFRP1^16^, and SEPP1^49^. Of these candidate targets, 6 mRNAs shown in underline were confirmed for their significantly down-regulated expression by quantitative PCR analysis (Table 4 and Supplementary Figure 7b).

**Figure 4:**
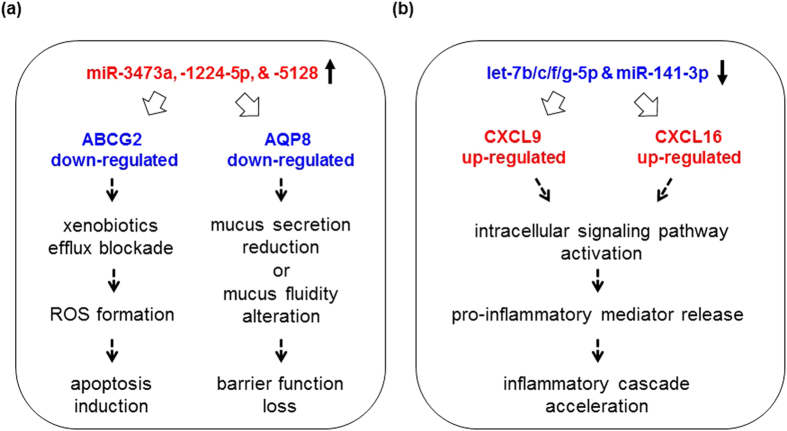
Proposed models for miRNA-initiated events occurred in inflamed large-IECs. (**a**) 3 miRNAs (miR-3473a, miR-1224-5p, and miR-5128) up-regulated significantly down-regulate AQP8 and ABCG2 expressions upon colitis. Reduction in AQP8 possibly in turn decreases secretion of mucus to alter its fluidity, resulting eventually in barrier function of large-IECs upon inflammation. Reduced ABCG2 allows cells to inhibit efflux of xenobiotics and is causative of IEC apoptosis via forming reactive oxygen species (ROS). (**b**) Reduction in let-7b/c/f/g-5p and miR-141-3p increases expression of putative chemokine targets, CXCL9 or CXCL16, in inflamed large-IECs. This augmentation activates intracellular signaling pathway to release pro-inflammatory mediators, causing further promotion of inflammatory cascades.

Based on the results from the correlations between miRNAs and their targets significantly altered selectively in inflamed large-IECs, we propose models for the intracellular pathways, which are initiated with significant alterations in miRNAs. The scenario is proposed in Fig. 4, in which alterations of cellular responses towards gut inflammatory cascades are associated with the miRNAs up- (a) or down-regulated (b).

In summary, through functional categorization of the target genes markedly up- or down-regulated in inflamed large-IECs, our study has revealed potentially important gene-regulation loops. As a result, the novel miRNAs revealed in the current study contribute to our integrated understanding of the miRNA-orchestrated manipulation of gene expression integral to the maintenance or disruption of gastrointestinal homeostasis.

## Methods

### Mice

Balb/c mice (age, 8–10 wk) were purchased from Clea Japan (Tokyo, Japan). All of the mice were maintained with free access to food and water on a standard 12-12 h light-dark cycle. Mice were acclimated for at least 1 wk before they entered the study. All experiments were performed according to the *Guidelines for Use and Care of Experimental Animals* and approved by the Animal Committee of the Institute of Medical Science, the University of Tokyo.

### DSS-induced experimental colitis

To induce experimental colitis, mice had free access to 3% DSS (w/v, MW = 36,000–50,000; MP Biomedicals, Santa Ana, California, USA) in drinking water for 8 days. Healthy control mice received DSS-free drinking water. Clinical symptoms, including body weight, rectal bleeding, and diarrhea, were monitored daily. Mice were euthanized on day 8, and their intestines were removed.

### Histologic analysis

To address the degree of inflammation, samples of intestinal tissues were fixed in 4% paraformaldehyde (Nacalai Tesque, Kyoto, Japan) for 24 h, embedded in paraffin, sectioned at 5  μm, stained with hematoxylin and eosin, and evaluated by light microscopy (model BX53, Olympus, Tokyo, Japan).

### IEC isolation and sorting

A standard protocol was used, with minor modifications, to isolate IECs. Briefly, the small and large intestines were harvested individually from 8 to 10 mice and rinsed extensively with RPMI-1640 (Gibco, Grand Island, New York, USA) after Peyer’s patches were removed (for small intestine). The rinsed intestines were opened longitudinally and macerated; the tissue pieces were shaken gently in RPMI-1640 containing 2 mM EDTA and 10% FCS. The tissue preparations were passed through 70-μm mesh filters, and the resulting single-cell suspensions were applied to Percoll (GE Healthcare, Little Chalfont, United Kingdom) density gradients of 25%, 40%, and 75%. After centrifugation at 2,000 × *g* for 20 min, the interface between the 25% and 40% layers was collected to obtain IECs. The cells were stained with antibodies to epithelial cell adhesion molecule (EpCAM) (Biolegend, San Diego, California, USA), or CD45 (Biolegend) and nucleic acid dye (Via-Probe) (Becton Dickinson, Franklin Lakes, New Jersey, USA). The EpCAM^+^ cells were further validated to be IECs via intracellular staining with antibody to pan-cytokeratin (Abcam, Cambridge, Massachusetts, USA) and Fix and Perm reagents (Invitrogen, Carlsbad, California, USA). The IECs were sorted (FACS Aria III, Becton Dickinson). Because the efficiency of this method for isolating highly purified (>98%) viable cells from gut tissues has been established previously in our laboratory, we used the sorted IECs immediately, without additional evaluation, in subsequent experiments. The data were analyzed by using FlowJo software (Ashland, Oregon, USA).

### Microarray analysis

The live IECs sorted from the intestines of 8 to 10 mice per group (untreated [normal] small intestine, untreated large intestine, DSS-inflamed small intestine, and DSS-inflamed large intestine) were pooled; total RNA was isolated from each pool by using Trizol (Invitrogen) and used to create microarrays to obtain their miRNA and mRNA expression profiles. Total RNA was labeled (3D-Gene Labeling Kit, Toray, Kamakura, Japan) according to the manufacturer’s instructions; labeled RNAs were hybridized onto 3D-Gene oligo chips for miRNA (approximately 1,900 genes mounted) or mRNA (approximately 24,000 genes mounted). The oligonucleotide sequences of the probes were confirmed by the miRBase miRNA database (http://microrna.sanger.ac.uk/sequences/). After stringent washes of the sample, fluorescent signals were scanned (3D-Gene Scanner, Toray) and analyzed by using 3D-Gene Extraction software (Toray). The relative expression level of a given miRNA or mRNA was calculated by comparing the signal intensities of valid spots throughout the microarray experiments^50^.

### Target prediction for miRNAs among IEC-expressed genes

The results obtained from miRNA and mRNA microarrays from viable IECs were applied to the public repository of putative miRNA–mRNA associations available from TargetScan^9^ to identify potential miRNA targets among expressed genes in IECs.

### Quantitative real-time PCR analysis

Total RNA was isolated from the sorted cells in succession by using Trizol (Invitrogen) as described earlier. cDNA was synthesized from approximately 800 ng to 1 μg of total RNA (NCode miRNA First-strand cDNA Synthesis and qRT–PCR (Invitrogen) or PrimeScript (Takara, Japan) kits and applied to quantitative real-time PCR analysis using a Power SYBR Green PCR Master Mix (Invitrogen). All reactions were repeated at least three times, and U6 and β-actin were used as endogenous controls to normalize the expression of miRNAs and mRNAs, respectively. Gene expression was analyzed by using a StepOnePlus Real-Time PCR System (Applied Biosystems, Foster City, California, USA). The forward primers for miRNAs were designed according to the manufacturer’s instructions (NCode Kit, Invitrogen), and the universal qPCR primer supplied in the kit was used as the reverse primer. The primer sequences used in this study are shown in Supplementary Table 1.

### Statistical analysis

The data are presented as mean ± 1 standard deviation for at least three independent experiments. Student’s *t*-test was used to compare the groups with control values (DSS-untreated mice).

## Discussion

We have shown here that the IECs in the large intestine contain numerous miRNAs that actively participate in inflammation. The putative networks among miRNAs and their targets in large-IECs from mice with DSS-induced colitis were nearly unique compared with those in similarly obtained small-IECs. Moreover, we disclosed several novel miRNAs that might modulate the expression of key molecules involved in either ameliorating or aggravating IBD pathology. Thus our study provides an initial step toward understanding, at the molecular level, the specific susceptibility of the large intestine to DSS-induced inflammation and identifying candidate miRNA biomarkers associated with either promotion or suppression of the inflammatory disorder.

It is worthy to focus on IECs in DSS-induced colitis model in two important aspects; alteration of barrier function and trigger of inflammatory stimulation. In the model the sulfated polysaccharide of DSS causes fatal injury of IECs and alter their mucosal barrier function^51^. Epithelial loss of barrier function results in inflow of microorganisms and their toxic products into lamina propria. This influx further stimulates to recruit innate and cytotoxic immune cells such as neutrophils or macrophages and secreting pro-inflammatory mediators^52^. In contrast, development of DSS-induced colitis is supposed to be independent of T cell-mediated adaptive immune responses^53^. Therefore, comprehensive analysis of IECs in the context of this colitis model will be fundamental in understanding their function as contributors of innate-immunity mediated gut inflammation.

It is generally recognized that the small and large intestines differ in their susceptibilities to gut inflammation and that environmental differences including resident microflora might be the root cause^54^. Due to their communication with microflora, IECs manifest biological responses to inflammatory stimuli such as invasive pathogens or chemical attack during IBD progression^55^. Because the biological responses in IBD can be revealed as alterations in gene expression or regulation, miRNAs represent a key regulatory component, primarily by reducing the levels of inflammatory or regulatory molecules involved in IBD^56^,^57^. Thus, to clarify the mechanisms underlying the differing sensitivity of the small compared with large intestine to IBD, acquiring comprehensive expression database of miRNAs and their target mRNAs that are expressed in the IECs of both tissues during normal or inflamed conditions is crucial.

The miR-223-3p was the only miRNA that showed increased expression in both inflamed small- and large-IECs in both microarray and quantitative PCR analyses (Supplementary Figure 6a and 6c). This finding is consistent with previous studies using clinical samples^58^. In contrast, our analysis of the targets of miR-223-3p by using TargetScan and microarray databases suggested that this miRNA regulates almost completely different targets between small- and large-IECs upon inflammation (Fig. 3 and Supplementary Figure 3a). However, miR-223-3p expression was greater in myeloid cells harvested from mouse large intestines than in similarly collected epithelial cells^11^. Evaluating the miR-223-3p expression of intestinal myeloid cells in the context of DSS-induced colitis is warranted in the near future.

The microarray data did not agree perfectly with the results from the quantitative PCR analysis, presumably due to technical differences between the methods^59^. For example, although miR-671-5p appeared to be down-regulated in inflamed small-IECs according to microarray data but augmented according to quantitative PCR, this pattern was mimicked for the results for this miRNA in the large-IECs (see Supplementary Figures 3b, 6a, and 6c). Thus, it seems acceptable to consider as biomarkers the miRNAs (as well as mRNAs) whose reproducible and consistent expression is validated in other analyses in addition to microarray (including quantitative PCR and Northern blotting). Because the miRNAs shown in Tables 3 and 4 showed reproducible expression in both microarray and quantitative PCR analyses, they might be considered as group biomarkers that are concomitantly down- (Table 3) or up-regulated (Table 4) by inflammatory stimuli in large-IECs. This point merits confirmation by other investigators.

Of the 18 mRNA targets up-regulated in inflamed large-IECs, 9 (CCL3, TREM1, NCF4, CXCL9, NCF2, SLC11A1, CD97, SFPQ, and CXCL16) were revealed to correlate with results from previous studies of IBD (Table 3)^17^,^18^,^19^,^20^,^21^,^22^,^23^,^24^,^25^. Moreover, the 20 mRNA targets included 5 genes for chemokines or cytokines (CCL3, CXCL9, IL17RA, IL4RA, and CXCL16) that have been associated with inflammatory conditions, including IBD^17^,^20^,^25^,^60^. This finding prompts us to postulate that some functional miRNAs repress these target genes at the posttranscriptional level under physiologic conditions. We suspect that those miRNAs would have been down-regulated during the inflammatory state and thereby unable to dampen the activity of their targets. We propose that the following miRNAs play a regulatory role in IBD: some of let-7-5p family, miR-21a-5p, miR-141-3p, miR-3963, and miR-3968. We noticed the only several miRNAs presumably involved in the regulation loops and the cooperativity and redundancy to regulate targets (Table 3). Therefore, microarray-based data analysis will be instrumental in identifying novel miRNA candidates that might suppress the activation of various inflammatory genes under normal conditions.

Among the 17 target genes down-regulated in inflamed large-IECs (Table 4), only 3 (17.6%) are known to be IBD-related: ABCG2 (ATP-binding cassette transporter G2), AQP8 (aquaporin 8), and SELENBP1 (selenium binding protein 1)^35^,^36^,^37^. ABCG2, whose expression is down-regulated in the IECs of patients with active IBD, is thought to help protect against various luminal threats^35^. Quantities of the water-channel protein AQP8 are decreased in cases of human IBD^36^. SELENBP1, a cellular antioxidant, is down-regulated in the colonic mucosa of patients with ulcerative colitis^37^. Together, these findings suggest to us that these three genes play a pivotal role in regulating IBD development in large-IECs. More importantly, three novel miRNAs—miR-1224-5p, miR-3473a, and miR-5128—likely suppress the expression of ABCG2, AQP8, or SELENBP1 presumably cooperatively (Table 4). Furthermore, miR-1224-5p, miR-3473a, and miR-5128 may represent biomarkers that are induced in large-IECs only under inflammatory conditions, whereas the same miRNAs, when down-regulated during the physiologic state, would be unable to repress key regulatory molecules (e.g., ABCG2, AQP8, or SELENBP1), thus maintaining gastrointestinal homeostasis. These hypothetical regulation prediction needs to be verified by examining transgenic *in-vivo* models that possess enterocyte-specific, overexpressed miRNAs in the near future. It is also pivotal to elucidate any clinical relevance of current study by analyzing intestinal samples from patients with ulcerative colitis or Crohn’s disease.

In conclusion, we expect that the miRNA database profile we obtained by using sorted IECs will be instrumental in determining, at the genetic and molecular levels, the source of the different susceptibilities of the small and large intestine to inflammation and their differences in inflammation-induced pathology. Whereas the expression of few miRNAs was significantly increased or decreased in small-IECs upon DSS-induced inflammation, large-IECs in the experimental IBD model exhibited markedly altered patterns (both up- and down-regulation) in the expression of mRNAs and miRNAs; our study may be the first to compare these genetic profiles between normal and inflamed IECs. Furthermore, this immense shift in the genetic components of large-, but not small-, IECs under the situation of IBD might represent the quality differences between both intestines. Although the biological significance of these biomarker candidates needs to be validated through *in vitro* and *in vivo* approaches, the current study is an initial step toward the creation of a global platform in the IEC miRNAs for understanding an implicate solution to identifying new biomarkers not only for miRNAs and mRNAs but also for their correlation loops. Finally, this work advances our understanding of how IECs rearrange their genetic components to cope with chaotic situations such as IBD progression.

## Additional Information

**How to cite this article**: Lee, J. *et al.* Profiles of microRNA networks in intestinal epithelial cells in a mouse model of colitis. *Sci. Rep.* **5**, 18174; doi: 10.1038/srep18174 (2015).

## Supplementary Material

Supplementary Information

## Figures and Tables

**Table 1:** Highly expressed miRNAs.

Increased in normal small-IECs (compared with normal large-IECs)							
miR-6243	43187	miR-1965	1840.2	miR-2136	548.1	miR-3067-3p	265.7
miR-5109	30926	miR-744-5p	1734.5	miR-709	545.1	miR-1895	263.8
miR-2137	29793	miR-6244	1730.4	miR-6378	504.7	miR-714	252.3
**miR-6240**	25457	miR-211-3p	1670.2	miR-296-5p	496.5	miR-486-3p	246.9
miR-5126	21891	miR-5128	1531.3	miR-92a-2-5p	492.2	miR-1934-3p	227.2
miR-3960	16884	miR-215-5p	1364.4	miR-365-1-5p	475.9	miR-363-5p	219
miR-2861	16171	miR-5621-5p	1356.8	miR-1893	457.8	**miR-31-5p**	218.5
**miR-711**	13240	miR-5130	1233.5	miR-671-5p	448.7	miR-5622-3p	215
miR-762	11201	miR-3473a	1144.9	**miR-1894-3p**	424.5	miR-6370	215
miR-6538	7735.3	miR-3107-3p	1144.5	**miR-6394**	402.9	miR-1249-3p	214.7
miR-328-5p	6137.8	miR-680	1117.6	**miR-6351**	399.2	miR-1940	202.2
miR-149-3p	4842.1	**miR-204-3p**	1102.8	miR-1249-5p	396.7	miR-6392-3p	170.8
miR-326-5p	3921	miR-3090-5p	1073.3	miR-705	380.6	miR-1198-5p	166.5
**miR-6236**	2569.2	miR-92b-5p	998.5	miR-6391	379	miR-223-3p	166.1
miR-5115	2450.3	miR-346-3p	987.4	miR-5122	356.9	miR-185-3p	158.8
miR-5627-5p	2341.9	miR-128-2-5p	949.4	miR-1964-5p	341.6	miR-3087-5p	146.8
miR-3473e	2248.7	miR-3104-5p	941.6	miR-5099	335.9	miR-712-3p	143.4
miR-3473b	2165.5	miR-5132-5p	870.5	**miR-5620-3p**	312.5	miR-615-5p	125.8
**miR-1892**	2066.9	miR-1224-5p	805.1	miR-5114	292.4	miR-710	125.2
miR-3072-5p	1982.1	**miR-370-3p**	652.1	miR-3474	290.1	miR-466f	112
miR-3102-5p	1979.3	**miR-5620-5p**	635.7	miR-6239	289.8		
miR-3077-5p	1893.9	miR-5116	572.3	miR-3572-5p	268.4		
**Increased in normal large-IECs (compared with normal small-IECs)**							
miR-6412	5600.3	let-7a-5p	577.9	miR-429-3p	496.4	**miR-378b**	362.6
**miR-200a-3p**	2073.9	**miR-141-3p**	570.2	**miR-27b-3p**	426.9	let-7g-5p	358
**miR-200b-3p**	1524.1	let-7f-5p	557.1	**miR-378a-3p**	416.6	**miR-103-3p**	343.6
miR-21a-5p	1137.5	**miR-29a-3p**	540.1	**miR-378d**	394.1	**miR-30d-5p**	258.1
**miR-23b-3p**	853.9	miR-30c-5p	537.5	miR-30b-5p	391.8	**miR-10b-5p**	255.3
**miR-23a-3p**	657.2	let-7d-5p	535.2	**miR-27a-3p**	390.9	miR-29b-3p	231
miR-125a-3p	630.9	miR-200c-3p	529.1	**miR-378c**	373.5	**miR-22-3p**	225.9
let-7c-5p	614.6	let-7b-5p	521.1	**miR-10a-5p**	369.4	miR-30a-5p	175
**miR-26a-5p**	608.4						
**Increased in inflamed small-IECs (compared with inflamed large-IECs)**							
**miR-6240**	18940	**miR-1892**	1897.3	**miR-5620-5p**	308.7	**miR-5620-3p**	245.5
**miR-711**	8050.7	**miR-204-3p**	485	**miR-1894-3p**	300.1	**miR-31-5p**	241.6
**miR-6236**	2438.7	**miR-370-3p**	419.7	**miR-6394**	298.1	**miR-6351**	220.9
miR-215-5p	2219.9						
**Increased in inflamed large-IECs (compared with inflamed small-IECs)**							
*miR-5126*	25994	**miR-5099**	1467.4	**miR-26a-5p**	668	**miR-27a-3p**	338.8
miR-690	18875	**miR-378a-3p**	1040.4	**miR-10a-5p**	663.6	miR-5112	305.5
miR-5105	15012	**miR-200b-3p**	1003.5	miR-107-3p	586.9	**miR-22-3p**	301
*miR-3473e*	5846.9	*miR-709*	977.1	miR-191-5p	562.9	miR-6349	251.7
*miR-3473b*	4691.6	**miR-378d**	914.5	*miR-3474*	508.4	**miR-27b-3p**	219.6
*miR-5128*	2937.8	**miR-23a-3p**	911.4	**miR-103-3p**	489.8	**miR-141-3p**	180.8
**miR-200a-3p**	2646.2	**miR-378c**	899.6	miR-770-3p	419.4	miR-712-5p	167.3
*miR-744-5p*	2513.9	**miR-23b-3p**	862.8	*miR-1249-5p*	406.4	miR-6406	139.4
*miR-3077-5p*	2129.2	**miR-29a-3p**	752.4	**miR-10b-5p**	380.2	miR-205-5p	131.4
*miR-5621-5p*	1518.1	**miR-378b**	685.3	**miR-30d-5p**	343.4		

The miRNAs above showed at least a 2-fold difference in expression as well as an increase in fluorescence intensity of at least 100 background-subtracted arbitrary units between compared groups and are listed in order of fluorescence intensity. Boldface indicates miRNAs whose expression was significantly increased above background in both the normal and inflamed conditions. Among the miRNAs that were increased in inflamed large-IECs compared with inflamed small IECs, the 11 miRNAs in italics overlapped with those increased in normal small-IECs, and the 10 miRNAs that are underlined were not represented among those highly expressed in either normal small- or large-IECs.

**Table 2:** Fold change (log base 2) in miRNA expression in inflamed compared with normal IECs.

Inflamed small-IECs (compared with normal small-IECs)							
**miR-223-3p**	1.327	miR-365-1-5p	−1.62	miR-3102-5p.2-5p	−1.317	miR-1249-5p	−1.07
		miR-375-5p	−1.53	miR-6391	−1.186	miR-5130	−1.049
miR-345-3p	−4.002	miR-5622-3p	−1.511	miR-204-3p	−1.185	miR-5620-5p	−1.042
miR-346-3p	−1.796	miR-5122	−1.5	miR-1983	−1.161	miR-6366	−1.036
miR-1893	−1.679	miR-1964-5p	−1.429	miR-671-5p	−1.122	miR-6378	−1.025
miR-141-3p	−1.667	miR-3960	−1.422	miR-5105	−1.12	miR-211-3p	−1.02
miR-1902	−1.652	miR-494-3p	−1.412	miR-3963	−1.108	miR-1965	−1.006
miR-1934-3p	−1.626	miR-5112	−1.384	miR-3077-5p	−1.073		
**Inflamed large-IECs (compared with normal large-IECs)**							
miR-3473e	3.499	miR-6406	2.153	**miR-690**	1.561	miR-2861	1.414
miR-5099	3.481	miR-6349	2.112	miR-326-5p	1.508	**miR-671-5p**	1.364
**miR-223-3p**	3.415	miR-5126	2.087	miR-149-3p	1.499	miR-378a-3p	1.32
**miR-5128**	3.339	miR-3077-5p	2.009	**miR-5116**	1.494	miR-378c	1.268
**miR-3473b**	3.048	miR-709	2.007	miR-3090-5p	1.485	**miR-3473a**	1.245
**miR-680**	2.828	**miR-1224-5p**	1.909	miR-5621-5p	1.477	miR-378d	1.214
miR-3474	2.484	miR-1249-5p	1.907	miR-191-5p	1.47	miR-107-3p	1.165
miR-6244	2.469	miR-705	1.882	miR-3104-5p	1.468	miR-3072-5p	1.141
miR-744-5p	2.443	miR-770-3p	1.682	miR-365-1-5p	1.461	miR-5109	1.13
miR-762	2.33	miR-3960	1.67	miR-6538	1.46	miR-1893	1.129
miR-328-5p	2.329	miR-6240	1.64	miR-92a-2-5p	1.45	miR-17-5p	1.066
miR-6243	2.304	**miR-5130**	1.576	miR-2137	1.449	miR-5132-5p	1.022
miR-6239	2.285	miR-714	1.562	miR-1965	1.443		

miR-125a-3p	−2.568	miR-6412	−1.683	**let-7f-5p**	−1.453	**let-7b-5p**	−1.062
miR-1902	−2.55	**let-7g-5p**	−1.666	miR-29b-3p	−1.412	**miR-21a-5p**	−1.051
miR-1983	−2.126	**miR-141-3p**	−1.657	**miR-3968**	−1.304	miR-3072-3p	−1.01
**miR-3963**	−1.774	miR-375-5p	−1.462	**let-7c-5p**	−1.14		

The miRNAs above showed at least a 2-fold difference in expression as well as an increase in fluorescence intensity of at least 100 background-subtracted arbitrary units between compared groups and are listed in order of the magnitude of fold change. Positive numbers indicate upregulation of expression and negative numbers indicate downregulation in inflamed IECs compared with normal IECs. Boldface indicates miRNAs for which the change in expression was confirmed by Q-PCR analysis.

**Table 3:**
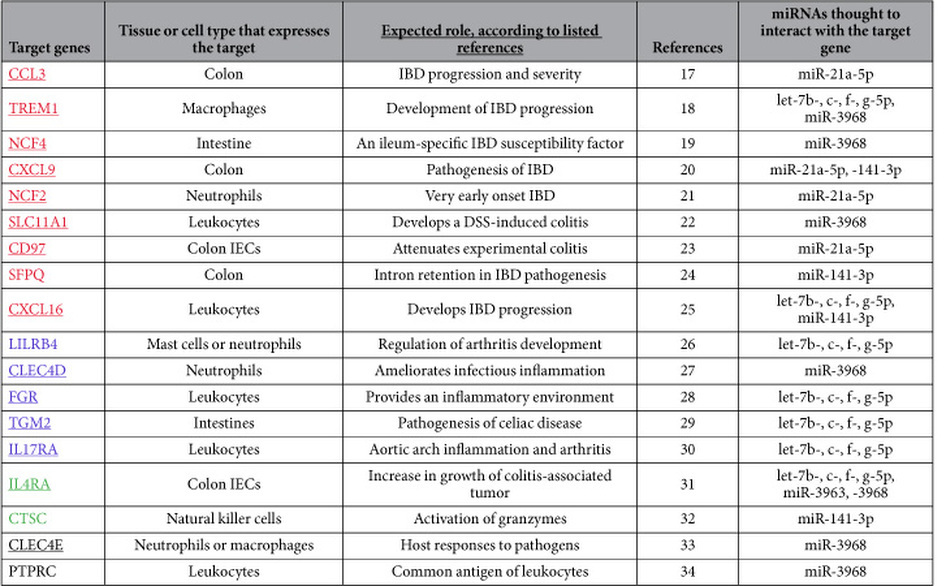
mRNA targets highly increased in inflamed large-IECs.

The mRNA expression of the target genes listed above was inverse to that of their corresponding miRNA(s); underlining indicates genes whose expression levels were confirmed by quantitative PCR analysis. In addition, target genes are organized into four categories according to the process in which their gene product is thought to function: IBD (red), inflammation other than IBD (blue), cancer (green), and gut immunity or homeostasis (black). The miRNAs shown are those whose altered expression was confirmed through both microarray and quantitative PCR analyses.

**Table 4:**
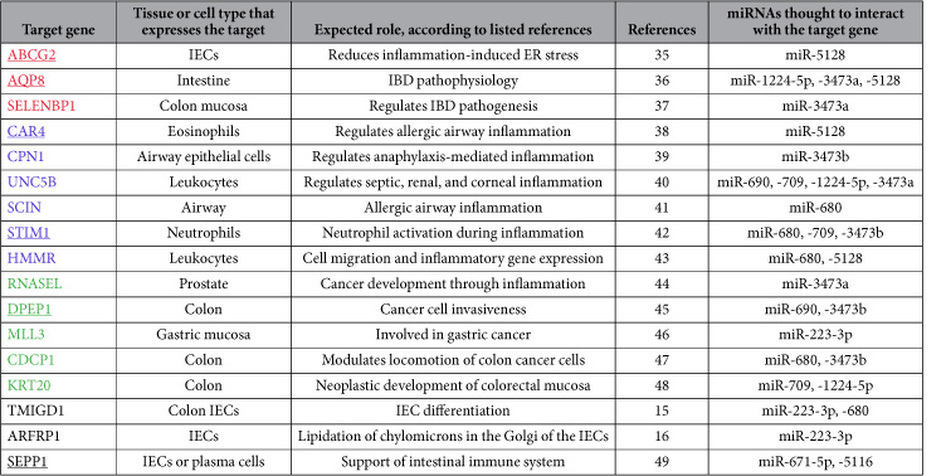
mRNA targets markedly reduced in inflamed large-IECs.

The mRNA expression of the target genes listed above was inverse to that of their corresponding miRNA(s); underlining indicates genes whose expression levels were confirmed by quantitative PCR analysis. In addition, target genes are organized into four categories according to the process in which their gene product is thought to function: IBD (red), inflammation other than IBD (blue), cancer (green), and gut immunity or homeostasis (black). The miRNAs shown are those whose altered expression was confirmed through both microarray and quantitative PCR analyses.
