# Supplementary material for: Profiles of microRNA networks in intestinal epithelial cells in a mouse model of colitis
Source: Sci Rep. 2015 Dec 9;5:18174. doi: 10.1038/srep18174 (PMC4673535; doi:10.1038/srep18174)
Supplement: Supplementary Information [file srep18174-s1.pdf]

Supplementary Information

Profiles of microRNA networks in intestinal epithelial cells in a mouse model of colitis

Juneyoung Lee[1,3,#], Eun Jeong Park[1,7,#], Yoshikazu Yuki[1], Shandar Ahmad[4], Kenji Mizuguchi[4], Ken J. Ishii[5,6], Motomu Shimaoka[7], and Hiroshi Kiyono[1,2,3,8,*]

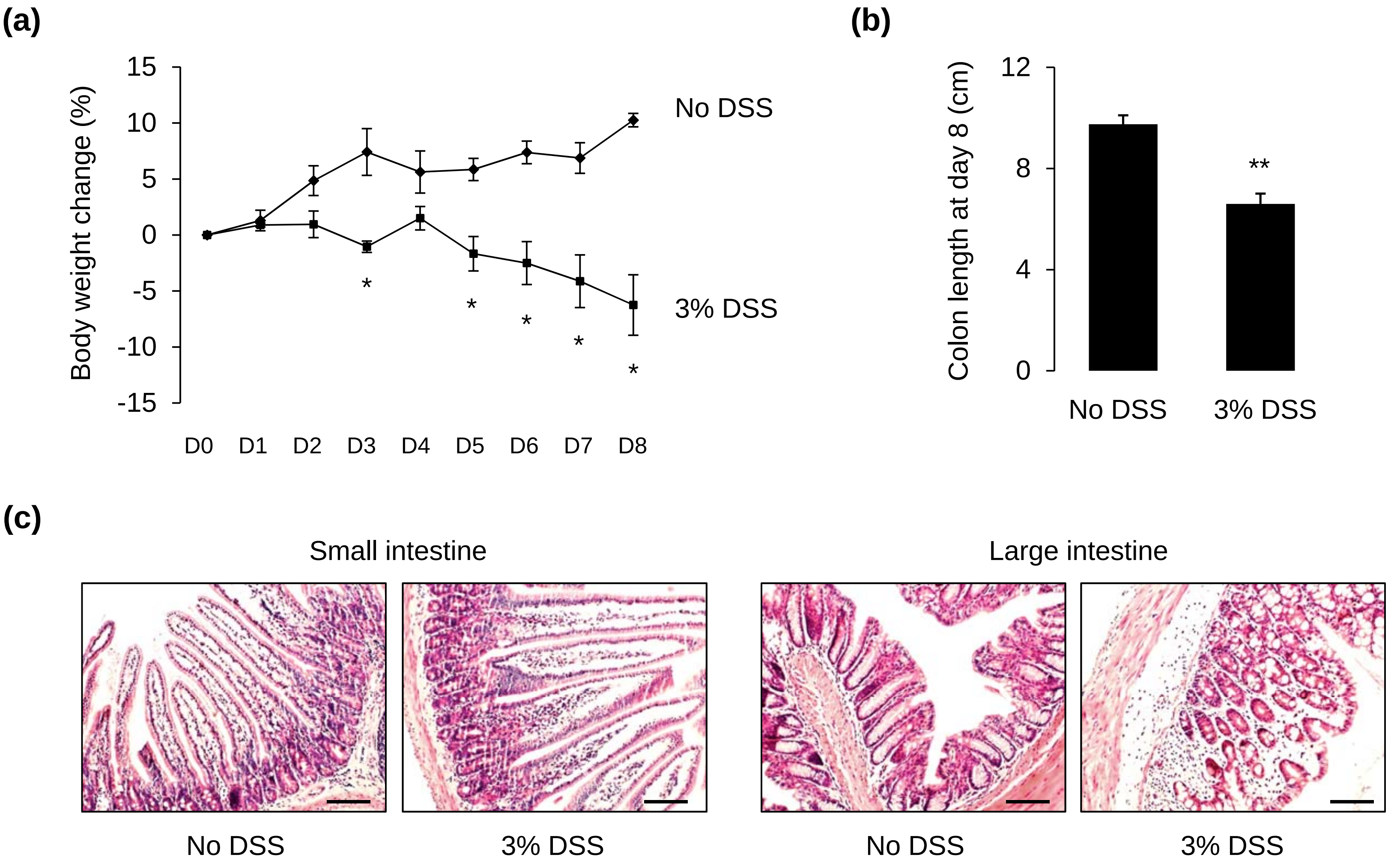

Supplementary Figure 1. Macroscopic and microscopic assessment of DSS-induced colitis. (**a**) The body weight of the mice treated with or without 3% DSS for 8 days was monitored once daily. Mice treated with 3% DSS showed a significant loss of body weight compared with the control (no DSS) group (n = 4 mice/group). (**b**) Colon lengths were measured. The mice treated with DSS exhibited a significant reduction in colon length (n = 4 mice/group). Data are presented as mean ± 1 standard deviations. *, 0.01 < p < 0.05; **, 0.001 < p < 0.01 vs. control (two-tailed Student's t-test). (**c**) The histology of intestinal tissues was examined in control and DSS-treated mice on day 8 by means of hematoxylin and eosin staining of paraffin-embedded sections. Scale bars, 100 μm. Data are representative of at least three independent experiments.

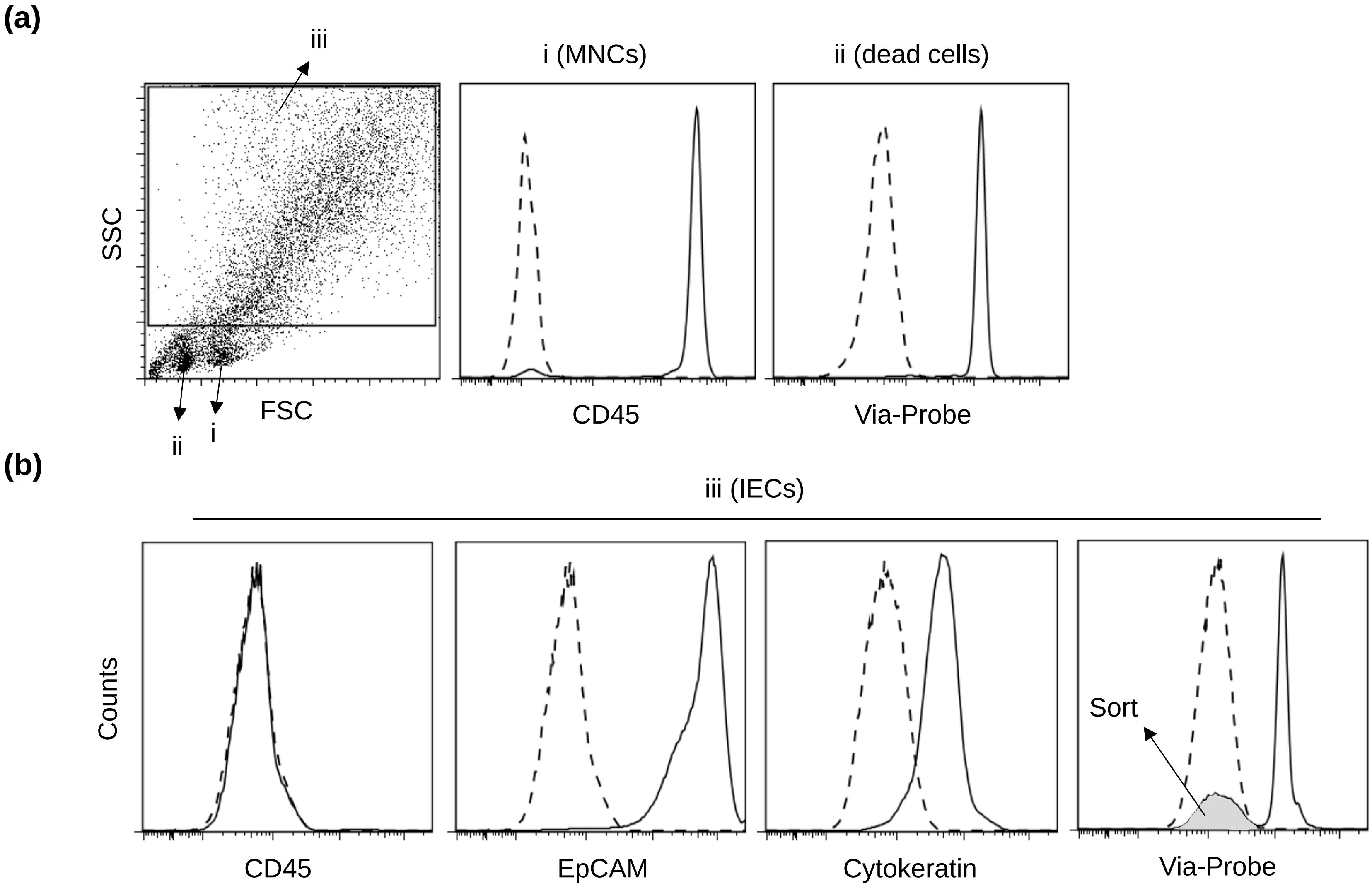

Supplementary Figure 2. Isolation of IECs. IECs were first isolated by a discontinuous density-gradient centrifugation from single-cell suspensions of small and large intestinal epithelium. The IECs isolated were then stained for cell viability (Via-Probe), gated by flow cytometry according to cell size, and sorted by fluorescence-activated cell sorting. (**b**) The cells gated were found to exclude CD45$^+$ mononuclear cells (e.g., lymphocytes) and Via-Probe$^+$ dead cells. (**b**) The IECs gated were CD45$^-$ and positive for epithelial cell adhesion molecule EpCAM$^+$ as well as for intracellular cytokeratin$^+$. Only live (Via-Probe$^-$) IECs were used in subsequent experiments. Histograms indicate representative results with the normal large-IECs. The IECs from all 4 samples (normal and inflamed small and large intestines) were isolated by the same methods.

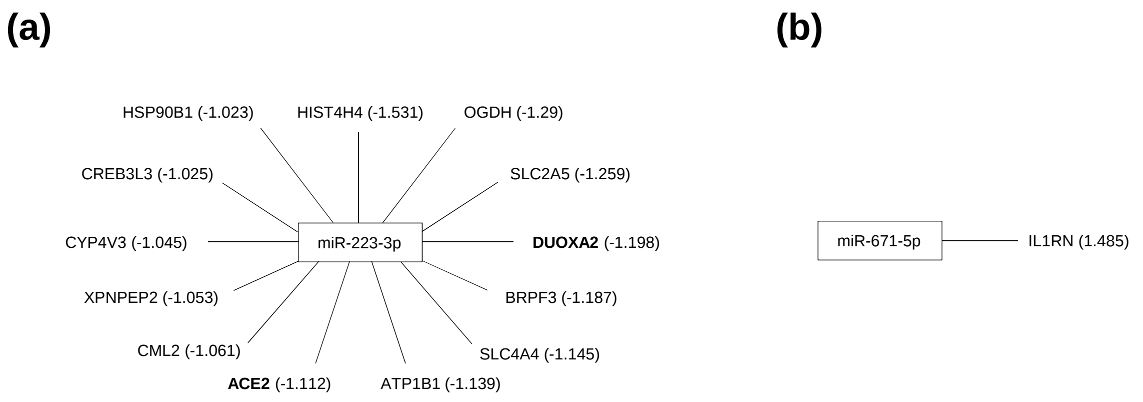

Supplementary Figure 3. The miRNA-regulated interactions in inflamed small-IECs. (**a**) Up-regulated miR-223-3p is associated with 13 target genes that were down-regulated in inflamed IECs. (**b**) In contrast, down-regulated miR-671-5p has a single target gene that was up-regulated in inflamed IECs. The numbers in parentheses indicate the fold-change (log base 2) of the genes in inflamed, compared with normal, IECs. The target genes are listed clockwise in order of their fold-change. Correlations were based on a TargetScan database analysis.

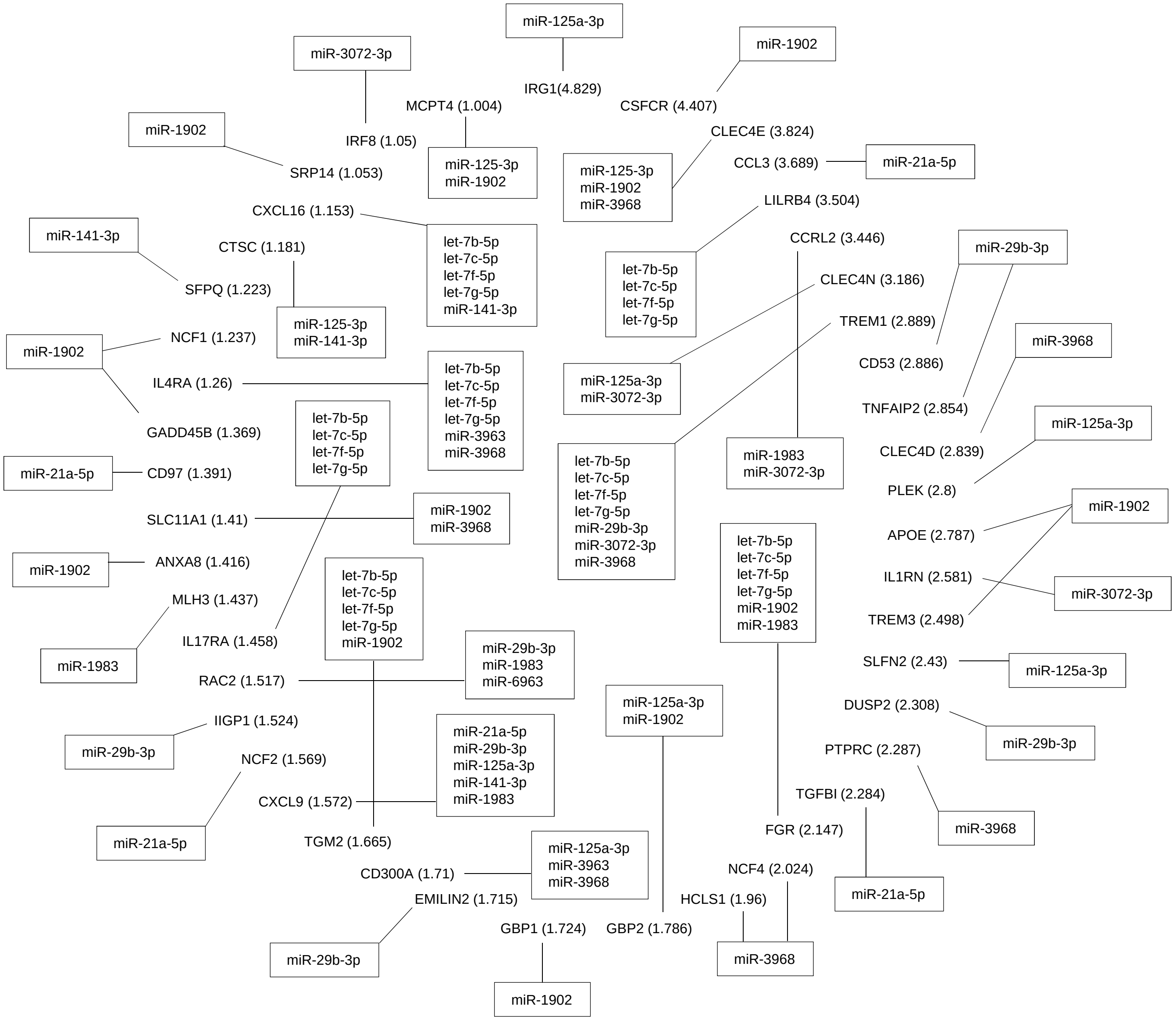

Supplementary Figure 4. The miRNA-regulated interactions in inflamed large-IECs. The predicted interactions between miRNAs and target genes in large-IECs were visualized according to mutually inversely correlated expressions. Potential miRNA–mRNA regulatory interactions were shown according to the criteria used in this study (>2-fold induction and fluorescence intensity >100 arbitrary units above background). 13 miRNAs down-regulated in inflamed IECs might suppress the expression of 45 mRNA targets under normal conditions.

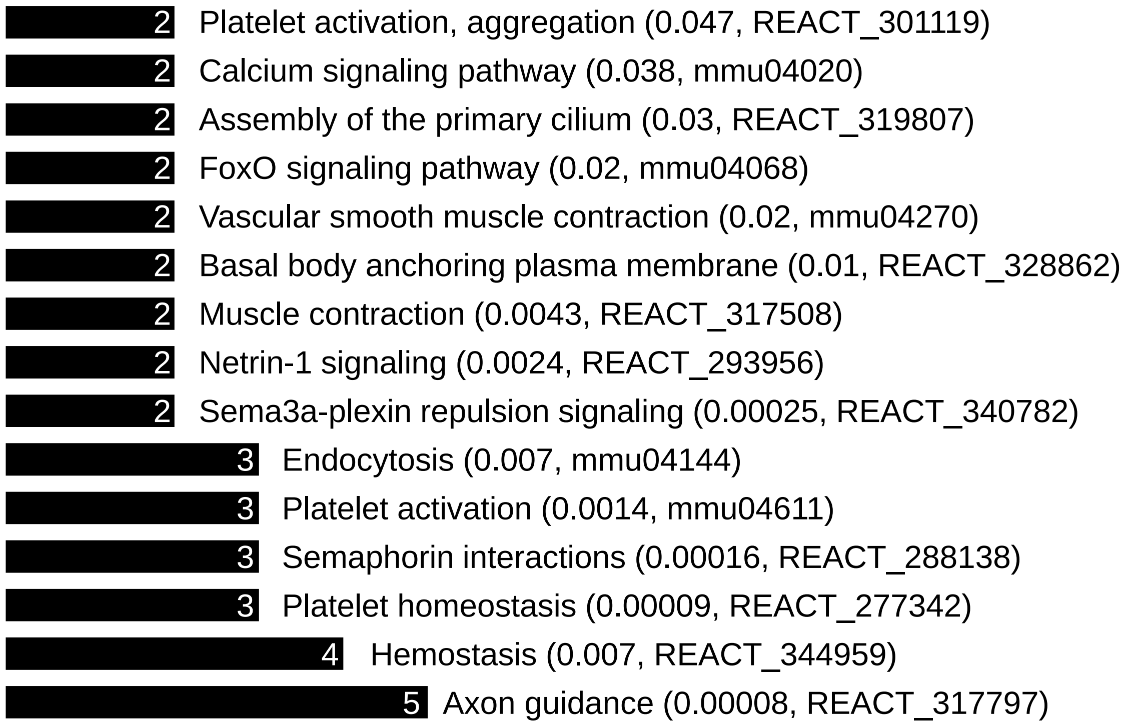

Supplementary Figure 5. Predicted biological pathways that are generated by analysis of the target genes of significantly altered miRNAs in inflamed large-IECs. TargetMine, which is a bioinformatics tools, was used to provide the pathway. The numbers in black bars indicate the number of different target genes associated in each pathway. Information inside the parenthesis shows *p*-values ($p < 0.05$) and corresponding source IDs.

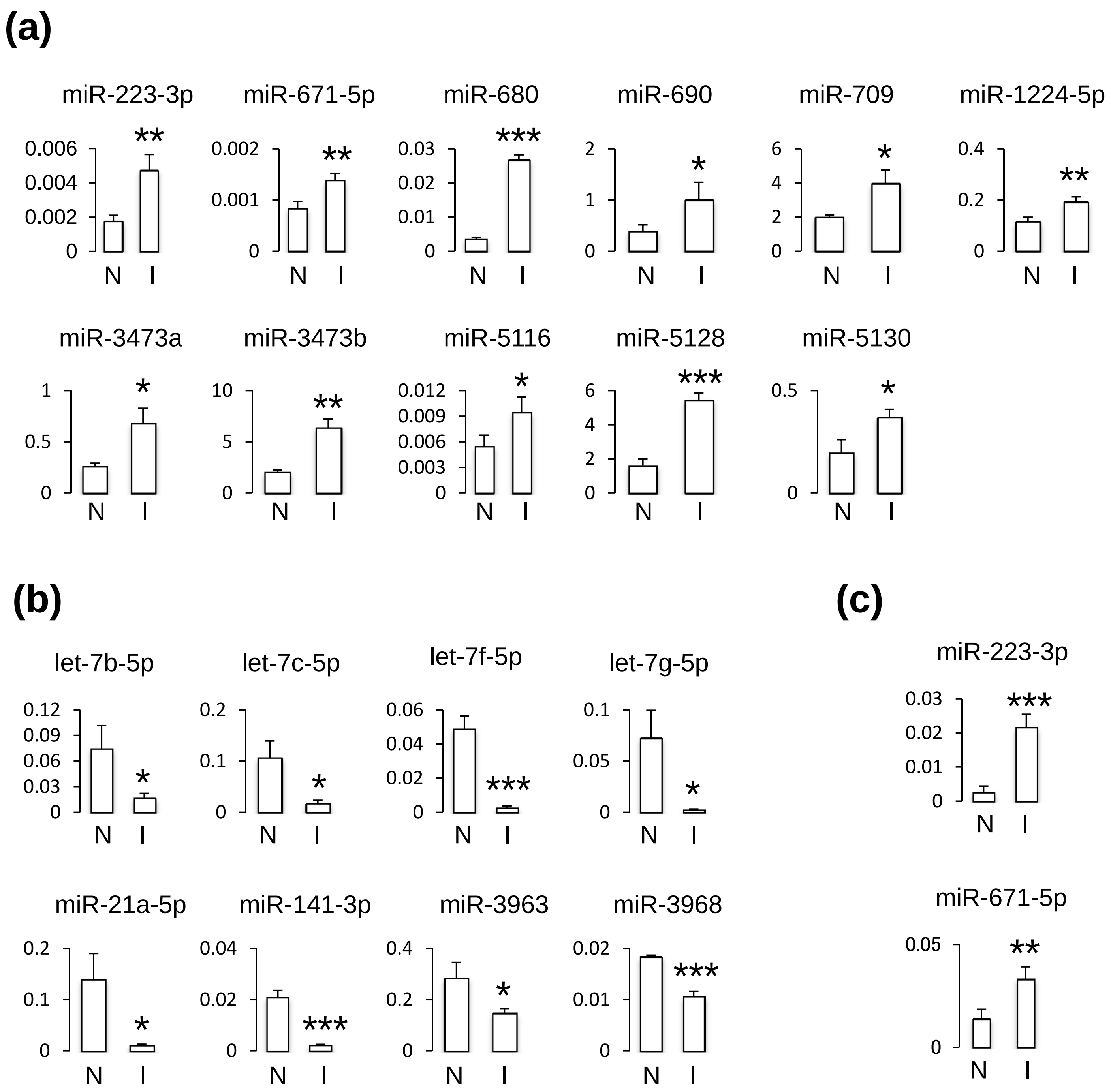

Supplementary Figure 6. Validation of miRNA expression by using quantitative real-time PCR. (**a**) These miRNAs showed up-regulated expression in inflamed (I), compared with normal (N), large-IECs, consistent with the microarray data. (**b**) These miRNAs showed down-regulated expression in the inflamed, compared with normal, large-IECs, consistent with the microarray data. (**c**) Expression of miR-223-3p was higher in inflamed, compared with normal, small-IECs; this finding is consistent with the microarray data. However, the expression of miR-671-5p was up-regulated in inflamed small-IECs, in contradiction of the microarray data (see Discussion). U6 was used as an endogenous control for normalizing miRNA levels. All experiments were repeated at least 3 times. The data are presented as mean $\pm$ 1 standard deviation. *, $0.01 < p < 0.05$; **, $0.001 < p < 0.01$; ***, $p < 0.001$ vs. control (two-tailed Student's *t*-test).

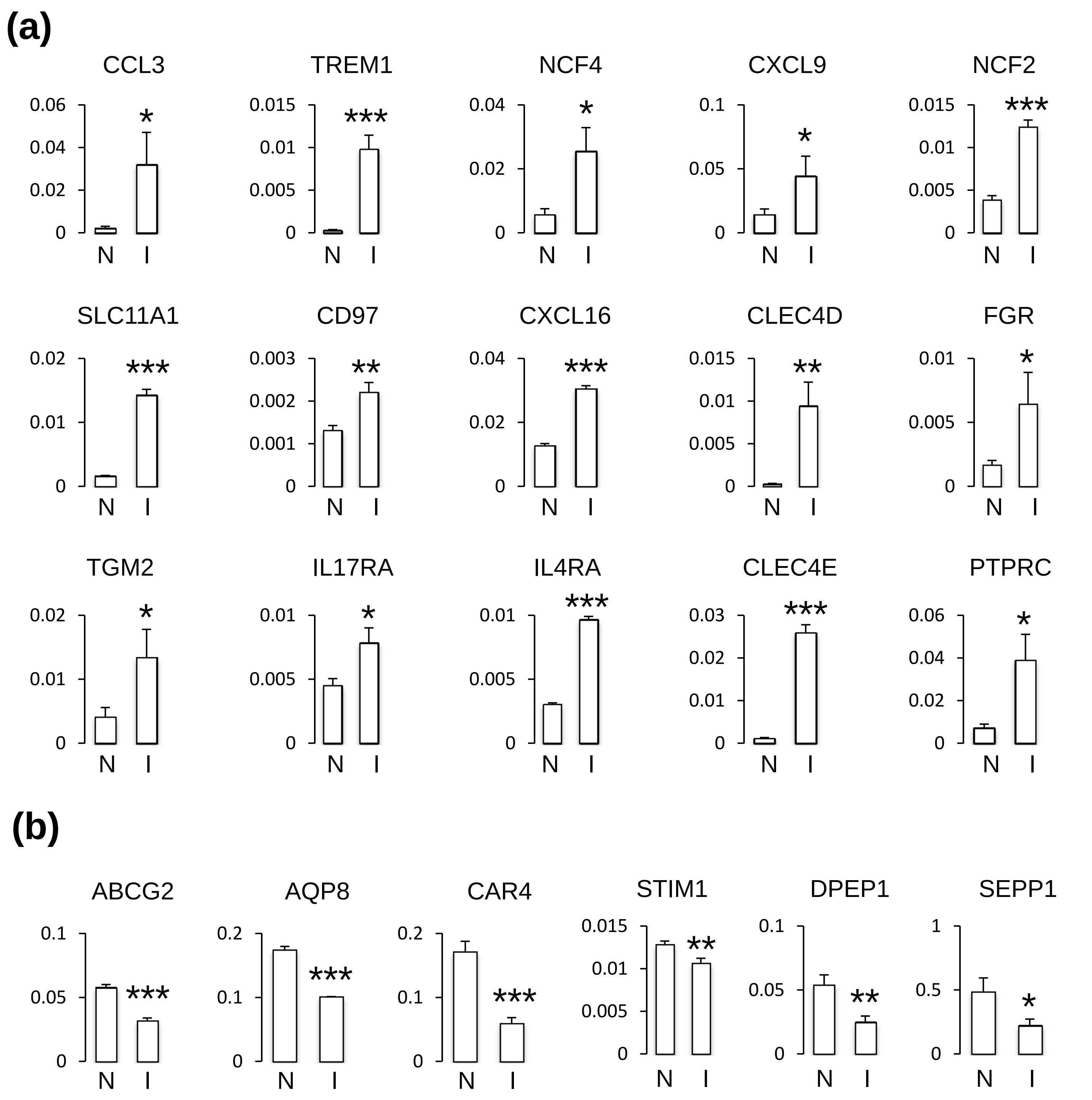

Supplementary Figure 7. Validation of mRNA expression by using quantitative real-time PCR. (**a**) These mRNAs showed up-regulated expression in inflamed (I), compared with normal (N), large-IECs, consistent with the microarray data. (**b**) These mRNAs showed down-regulated expression in the inflamed, compared with normal, large-IECs, consistent with the microarray data. b-actin was used as an endogenous control for normalizing mRNA levels. All experiments were repeated at least 3 times. The data are presented as mean ± 1 standard deviation. *, $0.01 < p < 0.05$; **, $0.001 < p < 0.01$; ***, $p < 0.001$ vs. control (two-tailed Student's *t*-test).

Supplementary Table 1. Primer sequences used in quantitative real-time PCR

	Forward (5′ → 3′)	Reverse (5′ → 3′)
let-7b-5p	TGAGGTAGTAGGTTGTGTGGTT	Universal primer
let-7c-5p	TGAGGTAGTAGGTTGTATGGTT	Universal primer
let-7f-5p	TGAGGTAGTAGATTGTATAGTT	Universal primer
let-7g-5p	TGAGGTAGTAGTTTGTACAGTT	Universal primer
miR-21a-5p	TAGCTTATCAGACTGATGTTGA	Universal primer
miR-141-3p	TAACACTGTCTGGTAAAGATGG	Universal primer
miR-223-3p	TGTCAGTTTGTCAAATACCCCA	Universal primer
miR-671-5p	AGGAAGCCCTGGAGGGGCTGGAG	Universal primer
miR-680	GGGCATCTGCTGACATGGGGG	Universal primer
miR-690	AAAGGCTAGGCTCACAACCAAA	Universal primer
miR-709	GGAGGCAGAGGCAGGAGGA	Universal primer
miR-1224-5p	GTGAGGACTGGGGAGGTGGAG	Universal primer
miR-3473a	TGGAGAGATGGCTCAGCA	Universal primer
miR-3473b	GGGCTGGAGAGATGGCTCAG	Universal primer
miR-3963	TGTATCCCACTTCTGACAC	Universal primer
miR-3968	CGAATCCCACTCCAGACACCA	Universal primer
miR-5116	TTTGATAGGAACCCCGCCTGA	Universal primer
miR-5128	CAATTGGGGCTGGCGAGATGGCT	Universal primer
miR-5130	CTGGAGCGCGCGGGCGAGGCAGGC	Universal primer
U6	GCGCGTCGTGAAGCGTTC	GTGCAGGGTCCGAGGT
ABCG2	CGCAGAAGGAGATGTGTT	TTGGATCTTTCCTTGCTGCT
AQP8	TGTGTAGTATGGACCTACCTGAG	ACCGATAGACATCCGATGAAGAT
CAR4	CTCCTTCTTGCTCTGCTG	GACTGCTGATTCTCCTTA
CCL3	TGAAACCAGCAGCCTTTGCTC	AGGCATTCAGTTCCAGGTCAGTG
CD97	CCTGGTCGGCGTGGAGAATGAAG	GGGCGATGGCGGTGATGGTC
CLEC4D	AGTAACGTGCATCCGAGAGG	GGAAGGCTCTCCAGCTAACA
CLEC4E	AGTGCTCTCCTGGACGATAG	CCTGATGCCTCACTGTAGCAG
CXCL9	TCCTTTTGGGCATCATCTTC	TTCCCCCTCTTTTGCTTTTT
CXCL16	CCTTGTCTCTGGCGTTCTTCC	TCCAAAGTACCCTGCGGTATC
DPEP1	ATGCGGTATCTGACCCTCAC	ATCTGCAAAGCGTCCTTCAT
FGR	TAAGATCCGAAAGCTGGACACG	CGACACCACCGCATACAGC
IL4RA	CACCTGGAGTGAGTGGAGTC	AGGCAAAACAACGGGATG
IL17RA	AGTGTTTCCTCTACCCAGCAC	GAAAACCGCCACCGCTTAC
NCF2	TCGGATTCACCCTCAGTCGCAG	GCATGTAAGGCATAGGCACGCTG
NCF4	CAAGGGTGTGTCTCCACAAG	TTGCTGTTCCCAGTGAAGTC
PTPRC	TCATGGTCACACGATGTGAAGA	AGCCCGAGTGCCTTCCT
SEPP1	TGTTGAAGAAGCCATTAAGATCG	CACAGTTTTACAGAAGTCTTCATCTTC
SLC11A1	GGACAGTTCGTGATGGAGGG	TTGAGTAGATCGTTGAGGCCG
STIM1	GCTCTCAATGCCATGCCTTCCAAT	TCTAGGCCATGGTTCAACGCCATA
TGM2	GCCTTGGAACTTTGGGCAGTTTGA	TCATCATTGCAGTTGACCATGCCG
TREM1	GAGCTTGAAGGATGAGGAAGGC	CAGAGTCTGTCACTTGAAGGTCAGTC
β-actin	CCTAAGGCCAACCGTGAAAAG	GTGCAGGGTCCGAGGT
